# PCR and real-time PCR primers developed for detection and identification of *Bifidobacterium thermophilum *in faeces

**DOI:** 10.1186/1471-2180-8-179

**Published:** 2008-10-10

**Authors:** Sophie Mathys, Christophe Lacroix, Raffaella Mini, Leo Meile

**Affiliations:** 1 Laboratory of Food Biotechnology, Institute of Food Science and Nutrition, ETH Zurich, Switzerland

## Abstract

**Background:**

Culture-independent methods based on the 16S ribosomal RNA molecule are nowadays widely used for assessment of the composition of the intestinal microbiota, in relation to host health or probiotic efficacy. Because *Bifidobacterium thermophilum *was only recently isolated from human faeces until now, no specific real-time PCR (qPCR) assay has been developed for detection of this species as component of the bifidobacterial community of the human intestinal flora.

**Results:**

Design of specific primers and probe was achieved based on comparison of 108 published bifidobacterial 16S rDNA sequences with the recently published sequence of the human faecal isolate *B. thermophilum *RBL67. Specificity of the primer was tested *in silico *by similarity search against the sequence database and confirmed experimentally by PCR amplification on 17 *Bifidobacterium *strains, representing 12 different species, and two *Lactobacillus *strains. The qPCR assay developed was linear for *B. thermophilum *RBL67 DNA quantities ranging from 0.02 ng/μl to 200 ng/μl and showed a detection limit of 10^5 ^cells per gram faeces. The application of this new qPCR assay allowed to detect the presence of *B. thermophilum *in one sample from a 6-month old breast-fed baby among 17 human faecal samples tested. Additionally, the specific qPCR primers in combination with selective plating experiments led to the isolation of F9K9, a faecal isolate from a 4-month old breast-fed baby. The 16S rDNA sequence of this isolate is 99.93% similar to that of *B. thermophilum *RBL67 and confirmed the applicability of the new qPCR assay in faecal samples.

**Conclusion:**

A new *B. thermophilum*-specific qPCR assay was developed based on species-specific target nucleotides in the 16S rDNA. It can be used to further characterize the composition of the bifidobacterial community in the human gastrointestinal tract. Until recently, *B. thermophilum *was considered as a species of animal origin, but here we confirm with the application of this new PCR assay the presence of *B. thermophilum *strains in the human gut.

## Background

Real-time quantitative polymerase chain reaction (qPCR) has recently emerged as promising tool for faecal microbiota monitoring in animal and human faeces [1-3] since culture-based methods are not suitable for quantification of certain microbial groups, species or strains in faeces [4]. Due to the role of bifidobacteria as probiotics much attention has been focused on the qPCR-based quantification of both the autochthonous bifidobacteria in faecal microbiota and on selected strains of bifidobacteria after consumption as probiotics [5-9]. Compared to fluorescence *in situ *hybridization (FISH), the most widely used method for culture independent quantification in faeces, qPCR is less developed in terms of the availability of specific probes [10]. On the other hand qPCR was shown to be about a 10 to 100 fold more sensitive than culture- and FISH-based enumeration techniques [11], as well as to be rapid, easy and more accurate for quantification of low levels of bacteria [12]. Several oligonucleotides were designed for the *Bifidobacterium *species found in the human intestinal tract, most of them based on the 16S rDNA sequence [11,13]. Other target sequences like the transaldolase encoding gene [5], heat-shock protein (HSP60) gene [14], intergenic spacer of the 16S-23S rRNA gene [15] are also being investigated for species-specific detection and quantification. Oligonucleotides targeting such sequences could also be used for developing qPCR primers.

*Bifidobacterium thermophilum*, being considered as an animal-associated commensal species, was never included in studies on the bifidobacterial composition of the human intestinal flora and to our knowledge, no oligonucleotide was designed for the development of *B. thermophilum*-specific PCR or qPCR assay until now. Recently, design of a pair of oligonucleotides for PCR amplification of a portion of the 16S rDNA of *B. thermophilum *was reported, but effective specificity of the assay was questioned [16]. Previously, we have isolated and characterized bifidobacteria with anti-*Listeria *activity from stool of newborns [17,18]. Strain RBL67 was identified as *B. thermophilum *using 16S rDNA sequence homology, comparative HSP60 sequence analysis, DNA-DNA genome hybridization and carbohydrate fermentation patterns [19]. This was the first demonstration of the presence of *B. thermophilum *in human faeces. In this study, we designed oligonucleotides specific for *B. thermophilum *that we used to develop PCR and qPCR assays to study the distribution of this species in human faecal samples. Finally, the qPCR technology let us isolate a strain of *B. thermophilum *from human infant faeces closely related to *B. thermophilum *RBL67.

## Results

### Design of a *B. thermophilum *specific PCR assay

Specificity of the *B. thermophilum *specific primer was assessed by PCR on colonies with primers btherm and the *Bifidobacterium *genus-specific primer lm3 (Table 1). Of the 17 *Bifidobacterium *and two *Lactobacillus *strains tested, positive signals (amplification of a fragment of approximately 1.5 kb) were only obtained with the three faecal isolates *B. thermophilum *RBL67, *Bifidobacterium thermacidophilum *subsp. *porcinum *RBL68 and RBL70 (Figure 2, lanes 1 to 3, respectively), *B. thermophilum *DSM20210^T ^(Figure 2, lane 6), and *B. thermacidophilum *subsp. *porcinum *LMG21689^T ^(Figure 2, lane 5).

**Table 1:** Oligonucleotides used in this study

**Oligonucleotide**	**Sequence 5'-3'**	**Reference**
btherm	GAT GTG CCG GGC TCC TGC ATG	This study
lm3	CGG GTG CTI CCC ACT TTC ATG	[20]
lm26	GAT TCT GGC TCA GGA TGA ACG	[20]
bak11w	AGT TTG ATC MTG GCT CAG	[21]
bak4	AGG AGG TGA TCC ARC CGC A	[22]
bthermRTF	TTG CTT GCG GGT GAG AGT	This study
bthermRTR	CGC CAA CAA GCT GAT AGG AC	This study
bthermTqM	*FAM-ATG TGC CGG GCT CCT GCA T-*TAMRA	This study
520F	CAG GAG TGC CAG CAG CCG CGG	[23]
520R	ACC GCG GCT GCT GGC	[23]
1100F	CAG GAG CAA CGA GCG CAA CCC	[23]
1100R	AGG GTT GCG CTC GTT G	[23]

*FAM (6-carboxyfluorescein): fluorescent reporter dye. TAMRA (6-carboxytetramethylrhodamine): quencher.

**Figure 1:**
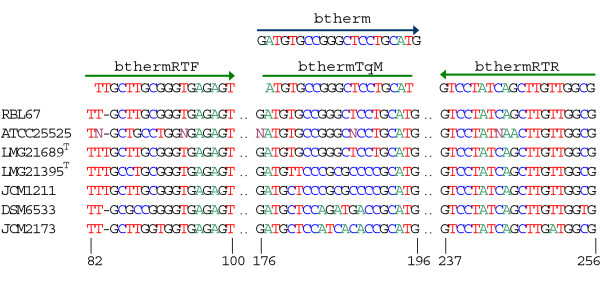
**Localization of the 16S rDNA targets for oligonucleotides designed in this study**. Multiple alignment of 16S rDNA sequences of *B. thermophilum *RBL67 [GenBank: DQ340557.1], *B. thermophilum *ATCC25525^T ^[GenBank: U10151.1], *B. thermacidophilum *subsp. *porcinum *LMG21689^T ^[GenBank: AY148470.1], *B. thermacidophilum *subsp.*thermacidophilum *LMG21395^T ^[GenBank: AB016246.1], *B. boum *JCM1211 [GenBank: D86190.1], *Bifidobacterium saeculare *DSM6533 [GenBank: D89330.1] and *B. breve *JCM1273 [GenBank: AF491832]. Numbers correspond to *E. coli *16S rDNA positions.

**Figure 2:**
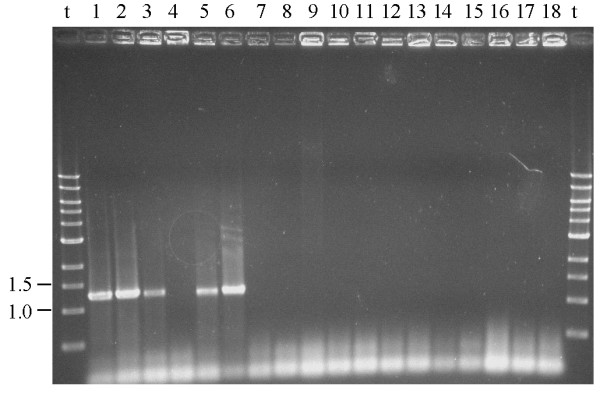
**Specificity of the PCR for *B. thermophilum *using primers btherm and lm3**. Agarose gel electrophoresis of PCR products (1.3 kb) obtained with primers btherm/lm3 on colonies of *B. thermophilum *RBL67 (1), *B. thermacidophilum *subsp. *porcinum *RBL68 (2), *B. thermacidophilum *subsp. *porcinum *RBL70 (3), *B. thermacidophilum *subsp. *thermacidophilum *LMG21395^T ^(4), *B. thermacidophilum *subsp. *porcinum *LMG21689^T ^(5), *B. thermophilum *DSM20210^T ^(6), *B. boum *DSM20432^T ^(7), *B. breve *DSM20213^T ^(8), *Bifidobacterium longum *NCC2705 (9), *Bifidobacterium coryneforme *DSM2026^T ^(10), *Bifidobacterium asteroides *DSM20089^T ^(11), *Bifidobacterium animalis *subsp.*lactis *DSM10140 (12), *B. animalis *subsp. *animalis *DSM20105 (13), *Bifidobacterium cuniculi *DSM20435^T ^(14), *Bifidobacterium adolescentis *DSM20083^T ^(15), *Bifidobacterium bifidum *DSM20456^T ^(16), *Lactobacillus delbrueckii *subsp. *lactis *DSM20072^T ^(17) and *Lactobacillus plantarum *subsp.*plantarum *DSM20174^T ^(18), t: Tridye 1-kb DNA ladder, in kb (New England Biolabs, Ipswich, MA, USA).

### Detection of *B. thermophilum *in faecal DNA samples by PCR

**Figure 3:**
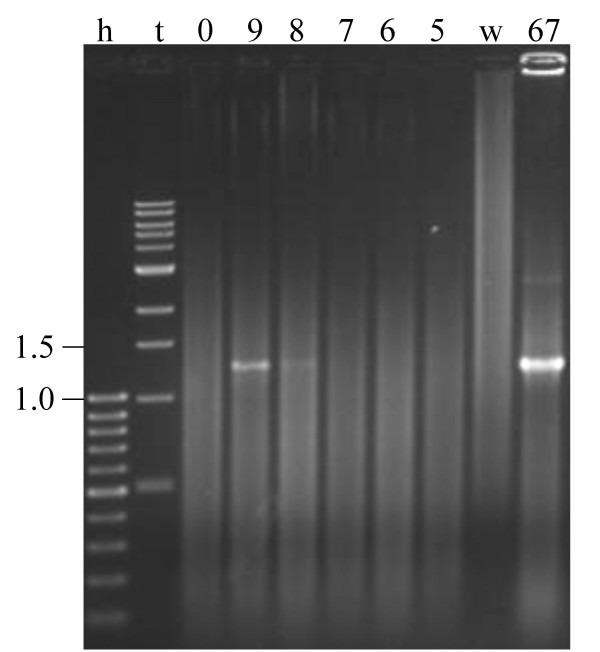
**Determination of the detection limit of the *B. thermophilum *PCR on faecal samples**. PCR amplification with primers btherm and lm3 of DNA isolated from faecal sample F0, the PCR-amplification negative control (0) spiked with 10^9 ^(9), 10^8 ^(8), 10^7 ^(7), 10^6 ^(6) or 10^5 ^(5) *B. thermophilum *RBL67 cells/g faeces; h: 100-bp DNA ladder [kb]; t: 1-kb DNA ladder [kb]; w: water; 67: PCR on a colony of *B. thermophilum *RBL67.

Classical PCR analysis with the *B. thermophilum *specific primers btherm and lm3 (Table 1) on total DNA isolated from faecal samples spiked with known quantities of *B. thermophilum *RBL67 showed that the detection limit of the method was 10^8 ^*B. thermophilum *cells per gram faeces (Figure 3). This high detection limit did not allow DNA amplification from any of the 17 faecal samples. Efficacy of PCR amplification on faecal DNA samples was confirmed by amplification of a 1.3 kb-DNA fragment from each faecal DNA sample generated with the *Bifidobacterium*-genus specific primer-pair lm26/lm3 [20] (data not shown).

### Development of a qPCR assay for detection of *B. thermophilum *in human faeces

The qPCR technology was chosen as an alterative to the classical PCR for its higher sensitivity. *B. thermophilum *specificity of qPCR performed with primers bthermRTF, bthermRTR and the TaqMan probe bthermTqM (Table 1) was tested by amplification of DNA isolated from six different *Bifidobacterium *strains belonging to four closely related species. A positive signal was obtained for *B. thermophilum *RBL67 (C_T _= 17.3 ± 0.5), *B. thermophilum *DSM20210^T ^(C_T _= 24.9 ± 0.3) and the closely related species *B. thermacidophilum *subsp. *porcinum *LMG21689^T ^(C_T _= 16.3 ± 0.4), but not for *B. thermacidophilum *subsp. *thermacidophilum *LMG21395^T^, *Bifidobacterium breve *DSM20213^T^, and *Bifidobacterium boum *DSM20432^T^. Amplification of DNA from *B. thermophilum *RBL67 with this new assay was shown to be linear for DNA concentrations ranging from 0.02 ng/μl (C_T _= 28.1 ± 0.3) to 200 ng/μl (C_T _= 15.3 ± 0.4) with a regression coefficient R^2 ^= 0.991 (data not shown).

### Screening of faecal samples by qPCR

**Figure 4:**
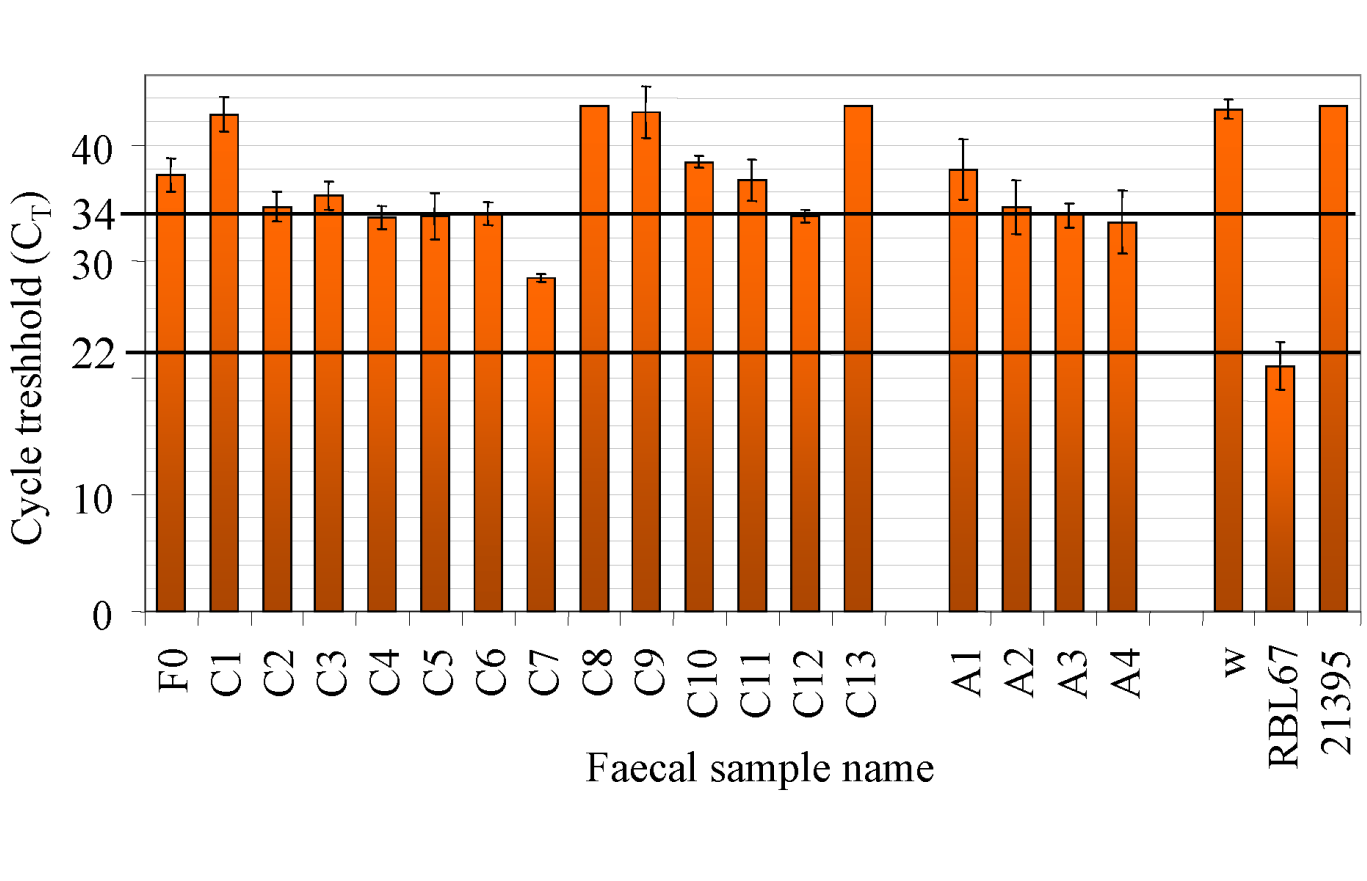
**Detection of *B. thermophilum *in faecal samples by qPCR targeting the 16S rDNA**. Cycle threshold (C_T_) values measured for faecal samples from 13 children (C1 to C13) and 4 adults (A1 to A4). F0: PCR-negative faecal sample, w: water instead of DNA, RBL67: DNA from pure culture of RBL67, 21395: DNA from *B. thermacidophilum *subsp. *thermacidophilum *LMG21395^T^. Values are means and standard deviations for three repetitions of the qPCR assay with three replicates each.

As for the classical PCR approach, the detection limit of the qPCR assay was determined by analysis of DNA isolated from spiked faecal samples and was equal to 1 × 10^5 ^bacterial cells per gram of faeces. Detection in faecal samples was shown to be linear between 1 × 10^9 ^and 1 × 10^5 ^cells per gram of faeces and corresponded to C_T _values comprised between 21.6 ± 0.6 and 33.8 ± 0.1, respectively, with a regression coefficient R^2 ^= 0.995 (data not shown). One of the 17 faecal samples (sample C7 from a 6 month-old breast-fed baby) gave a positive signal within this range (Figure 4) with a C_T _value of 28.6 ± 0.3, corresponding to a concentration of 5 × 10^6 ^cells per gram faeces.

### Application of the newly developed qPCR assay for faecal samples

Raffinose-*Bifidobacterium *(RB) medium and MRSC-NNLP were tested for isolation of new *B. thermophilum *strains from human faeces. MRSC-NNLP was chosen for isolation of bifidobacteria because it allowed a better growth of RBL67 (1.5 × 10^9 ^cfu/ml after three days of incubation at 40°C, in comparison to 2.9 × 10^7 ^cfu/ml in RB-medium and under the same conditions) and was easier to prepare. Approximately 1–5 × 10^8 ^colonies per gram faeces could be cultivated from all of the 17 samples plated on MRSC-NNLP agar. Microscopic observations of the isolates showed that the medium was not completely selective, allowing for the growth of non-rod-shaped microorganisms. 60 rod-shaped microorganisms were selected for PCR analysis on colony. Twenty-five of them were positive with the *Bifidobacterium *genus specific primers lm26/lm3, but only one of them gave a positive signal with btherm/lm3. This isolate, F9K9 from a 4-month old breast-fed baby, was streaked several times on MRSC agar and the absence of contaminants other than *Bifidobacterium *was confirmed by three PCR reactions with lm26/lm3, btherm/lm3 and bak4/bak11w [24] (data not shown). Sequencing of the 16S rDNA fragment amplified with lm26 and lm3 yielded a 1454-bp sequence which was 99.93% identical to the 16S rDNA of *B. thermophilum *RBL67. Sequence identities with other *Bifidobacterium *strains are summarized in Table 2.

**Table 2:** 16S rDNA sequence identities of isolate F9K9 with published 16S rDNA sequences

**Accession**	**Description**	% I*
DQ340557.1	*B. thermophilum *RBL67 [19]	99.93
AY148470.1	*B. thermacidophilum *subsp. *porcinum *LMG21689^T ^[28]	99.64
D86190.1	*B. boum *JCM1211 (DSM20432^T^)	98.15
AB016246.1	*B. thermacidophilum *subsp.*thermacidophilum *LMG21395^T ^[27]	97.02
U10151.1	*B. thermophilum *ATCC25525^T ^(DSM20210^T^)	95.82
D89330.1	*B. saeculare *DSM6533	95.29
AF491832	*B. breve *JCM1273 (= DSM20091)	95.16

*The percentage of identity (% I) was determined by comparison of the sequence of F9K9 against the sequences present in the database with the BLAST tool from NCBI.

## Discussion

Real-time PCR (qPCR) is known to be a more sensitive technique than classical PCR. This is reflected by our results for specific amplification of 16S rDNA from spiked faecal samples, where changing from classical PCR to qPCR for the detection of *B. thermophilum *in faecal samples lowered the detection limit of the assay from 10^8 ^to 10^5 ^cells per gram faeces. The high sensitivity obtained for qPCR in this study is similar to detection limits reported by different groups for other *Bifidobacterium *species or genus specific qPCR assays. Matsuki *et al. *[11], Penders *et al. *[6] and Gueimonde *et al. *[25], for example, reported detection limits of 10^6^, 5 × 10^3 ^and 5 × 10^4 ^cells of *Bifidobacterium *spp. per gram faeces, respectively. The application of a recent qPCR technology using rRNA as target molecule combined with reverse transcriptase could further enhance the sensitivity down to 10^3 ^cells/g faeces [26]. This methodology could also be developed for the detection of other subdominant faecal bacteria such as *B. thermophilum*.

We have developed a qPCR assay which is specific for *B. thermophilum *although the assay is also positive for the type strain of *B. thermacidophilum *subsp.*porcinum*, and *B. thermacidophilum *subsp.*porcinum *RBL68 and RBL70 (these subspecies were originally named "*suis" *which is a synonym for "*porcinum*"), but not with *B. thermacidophilum *subsp. *thermacidophilum *(LMG21395^T^) [27]. Based on our published data including DNA-DNA genome hybrizations [19] we underline that *B. thermacidophilum *subsp. *porcinum *[28] should belong to the *B. thermophilum *species and consequently, we conclude that our qPCR system is specific for *B. thermophilum*.

Until now, *B. thermophilum *was considered as an animal-associated species, mainly present in faeces of ruminants and pigs. The amplification of a specific 16S rDNA sequence with our qPCR on the children faecal sample C7 as well as the isolation of a *B. thermophilum *isolate from children faeces during this work support the assumption of von Ah *et al. *[19] that presence of *B. thermophilum *in food cannot be used to discriminate between animal and human bacterial contamination, as previously suggested [29].

## Conclusion

This is the first report of the development of a qPCR assay for specific detection of *B. thermophilum*, a species that was not included in analysis of the composition of the bifidobacterial human intestinal microbiota until now. Using this assay, we detected *B. thermophilum *at a concentration of 5 × 10^6 ^cells per gram in one faeces sample, confirming the presence of this species in human faecal material.

## Methods

### Bacterial strains and culture conditions

Unless otherwise indicated, bifidobacteria and lactobacilli were grown in liquid cultures overnight in 10 ml MRSC medium consisting of MRS [30], obtained from Biolife (Milan, Italy) and supplemented with 0.05% L-cysteine hydrochloride, or on MRSC-agar plates (MRSC supplemented with 1.5% w/v agar). Incubation was carried out for 24 h at 37°C in anaerobic jars with an anaerobic atmosphere generation system (Oxoid AnaeroGen TM, Basel, Switzerland). *B. thermophilum *RBL67, as well as *B. thermophilum *subsp. *porcinum *RBL68 and RBL70 are human infant faecal isolates [17-19,31]. All of the other strains are commercial strains from DSMZ (German collection of microorganisms and cell cultures, Braunschweig, Germany) or LMG (Laboratories for Microbiology and Microbial Genetics, Ghent, Belgium).

### Isolation of bifidobacteria from faecal samples

Seventeen faecal samples from human adults (4) and breast-fed children between 1 to 6 months (13) were collected as already described [32]. Subjects or parents of the subjects were informed orally and in writing about the aims and procedures of the study and consent was obtained from them. The study protocol was reviewed and approved by the Ethical Committees of the canton of Zurich and the SPUK-committee of the University Children's Hospital of Zurich (project StV31/05).

For efficient growth of *B. thermophilum *strains from faecal samples, Raffinose-Bifidobacterium (RB) [33] and MRSC-NNLP [34] media were compared. Serial 10-fold dilutions of overnight cultures of *B. thermophilum *RBL67 (containing approximately 10^9 ^cfu/ml) in saline solution (8.5 g/Liter NaCl, 1 g/Liter peptone, 0.05% cysteine-HCl, pH 6–7) were plated on RB and on MRSC-NNLP, incubated for 3 days anaerobically at 40°C and cell counts were determined. Incubation temperature of 40°C was chosen as an additional selective condition, due to the relative heat tolerance of *B. thermophilum *spp. [19]. For isolation of bifidobacteria from faecal samples, 20 mg of samples were homogenized by vigorous vortexing in 0.2 ml of saline solution, 10-fold serially diluted in the same solution and spread on MRSC-NNLP agar plates. Plates were incubated for 3 days under anaerobic conditions at 40°C and single isolates were observed under light microscope. Rod-shaped bacteria were selected for further analysis.

### DNA purification methods

Total DNA was isolated from pure cultures of *B. thermophilum *RBL67, *B. thermophilum *DSM20210^T^, *Bifidobacterium thermacidophilum *subsp. *porcinum *LMG21689^T^, *B. thermacidophilum *subsp. *thermacidophilum *LMG21395^T^, *Bifidobacterium breve *DSM20213^T ^and *Bifidobacterium boum *DSM20432^T ^according to Leenhouts *et al. *[35]. Total DNA was prepared from 200 mg of 17 faecal samples using the QiAamp DNA Stool Mini kit (Qiagen, Basel, Switzerland) according to manufacturer's instructions. A PCR-amplification negative faecal sample (F0) was prepared by autoclaving twice one of the samples. For determination of the detection limit, 10-μl aliquots of F0 were spiked before DNA preparation with a 10-fold serial dilution of *B. thermophilum *RBL67 (overnight culture in MRSC) at concentrations ranging from 10^9 ^to 10^1 ^bacterial cells per g faeces. The extracted DNA was stored at -20°C.

### DNA sequencing, PCR and qPCR reactions

Primers and probe used in this study were synthesized by Microsynth and are listed in Table 1. The TaqMan probe bthermTqM was labeled with 5'-FAM as fluorescent reporter dye and 3'-TAMRA as quencher. Classical PCR was performed either on 2 μl DNA prepared from faecal samples as described above, or on 40 μl cell suspensions. For that, one colony was picked from an agar plate and resuspended in 210 μl of sterile, double distilled water. A 50-μl classical PCR reaction consisted of 2.5 U EuroTaq-DNA-Polymerase (Digitana, Horgen, Switzerland), 1.5 mM magnesium chloride (Digitana), 0.2 mM dNTP's (GE Healthcare) and 0.5 μM of each primer. When DNA isolated from faecal samples was used as template, 0.1 μg/ml BSA was added to the PCR reaction. Amplification conditions were as follows: 3 min at 95°C, 40 cycles of 15 sec at 95°C, 30 sec at 62°C and 2 min at 72°C, followed by 7 min at 72°C. Sequencing of the PCR product for 16S rDNA was performed by Microsynth (Balgach, Switzerland) using the primers btherm, 520F, 520R, 1100F, 1100R and lm3 (Table 1).

The qPCR reactions were set in a total volume of 25 μl, containing 2.5 μl of DNA extracted from faecal samples with the stool kit as described above, 12.5 μl of qPCR MasterMix from Eurogentec (Seraing, Belgium), 0.3 μM of each primer and 0.1 μM of the TaqMan probe. Reactions were run on an ABI PRISM 7700 Sequence Detector (Applied Biosystems, Rotkreuz, Switzerland). The amplification conditions were 2 min at 50°C, 10 min denaturation at 95°C, followed by 45 cycles of 15 sec at 95°C and 1 min at 60°C. The cycle threshold (C_T_), corresponding to the number of cycles after which the target-DNA concentration increase becomes exponential, was monitored. Results were analyzed using the SDS 2.1 Software (Applied Biosystems). All reactions were done in triplicate and repeated at least twice (three times when faecal DNA extract was used as a template). Values in the text are mean ± SD.

### Design of a *B. thermophilum *specific primer and qPCR assay

One hundred and eight sequences of bifidobacterial 16S rDNA of more than 1 kb in length were retrieved from the EMBL nucleotide sequence database  and were used, together with the sequence of the 16S rDNA of *B. thermophilum *RBL67 [GenBank: DQ340557], to prepare a multiple alignment with ClustalW in the sequence analysis program BioEdit [36]. The primer btherm (Table 1) was manually designed in a variable region. Specificity of the primer was verified *in silico *with FASTA and the BLAST program from NCBI (version 2.2.15). For *B. thermophilum*-specific PCR amplification, primer btherm was used together with the *Bifidobacterium*-genus-specific reverse primer lm3. Cell suspensions of *B. thermophilum *RBL67 and DSM20210^T^, *B. thermacidophilum *strains RBL68, RBL70, LMG21395^T ^and LMG21689^T^, *B. boum *DSM20432^T^, *B. breve *DSM20213^T^, *B. longum *DSM20219^T^, *B. coryneforme *DSM2026^T^, *B. asteroides *DSMZ20089^T^, *B. animalis *subsp. *lactis *DSM10140, *B. animalis *subsp. *animalis *DSM20105, *B. cuniculi *DSM20435^T^, *B. adolescentis *DSM20083^T^, *B. bifidum *DSM20456^T^, *Lactobacillus delbrueckii *subsp. *lactis *DSM20072^T ^and *Lactobacillus plantarum *subsp. *plantarum *DSM20174^T ^were used as template for amplification with btherm and lm3 under the conditions described below to test the specificity of the PCR. For the development of the qPCR assay, the btherm primer was modified to fit the lower melting temperature required for a TaqMan probe and adequate adjacent forward and reverse primers were designed with the program Primer3 [37] (Table 1). Aliquots of 5 μl of DNA (20 ng/μl) isolated from pure cultures of *B. thermophilum *RBL67, *B. thermophilum *DSM20210^T^, *B. thermacidophilum *subsp. *porcinum *LMG21689^T^, *B. thermacidophilum *subsp. *thermacidophilum *LMG21395^T^, *B. breve *DSM20213^T ^and *B. boum *DSM20432^T ^were amplified with this assay to assess its specificity. Localization of the primers btherm, bthermRTF and bthermRTR and of the TaqMan probe bthermTqM on an alignment of 16S rDNA sequences of seven bifidobacteria is shown in Figure 1.

## Authors' contributions

SM participated in the study conception and coordination, drafted the manuscript and designed the specific oligonucleotides. RM performed part of the experiments. LM and CL provided guidance during all parts of the work. All authors read and approved the final manuscript.
